# Involvement of caveolin-1 in skin diseases

**DOI:** 10.3389/fimmu.2022.1035451

**Published:** 2022-11-30

**Authors:** Naoko Takamura, Yukie Yamaguchi

**Affiliations:** Department of Environmental Immuno-Dermatology, Graduate School of Medicine, Yokohama City University, Yokohama, Japan

**Keywords:** caveolin-1, skin, aging, psoriasis, fibrosis, skin cancer, skin infection

## Abstract

The skin is the outermost layer and largest organ in the human body. Since the skin interfaces with the environment, it has a variety of roles, including providing a protective barrier against external factors, regulating body temperature, and retaining water in the body. It is also involved in the immune system, interacting with immune cells residing in the dermis. Caveolin-1 (CAV-1) is essential for caveolae formation and has multiple functions including endocytosis, lipid homeostasis, and signal transduction. CAV-1 is known to interact with a variety of signaling molecules and receptors and may influence cell proliferation and migration. Several skin-related disorders, especially those of the inflammatory or hyperproliferative type such as skin cancers, psoriasis, fibrosis, and wound healing, are reported to be associated with aberrant CAV-1 expression. In this review, we have explored CAV-1 involvement in skin physiology and skin diseases.

## 1 Introduction

The skin is the outermost layer and the largest organ in the human body. It consists of three layers: the epidermis, dermis, and skin-associated adipose tissue (1). The skin-associated adipose tissue includes dermal and subcutaneous adipocytes. Moreover, although these two layers are not physically distinguished in humans, they are likely functionally different. The epidermis is the outermost of the three layers, primarily consisting of keratinocytes, melanocytes, immune cells, and Merkel cells (2). The dermis is the layer beneath the epidermis, and its main components are non-cellular connective tissue collagen, elastic fibers, and the extracellular matrix (ECM), as well as cellular components, fibroblasts, macrophages, and mast cells (3). The dermis also contains skin appendages, such as hair follicles, sebaceous glands, and sweat glands, which are important components of the skin (3). Adipocytes are the prominent cells in the adipose tissue and are derived from mesenchymal fibroblast precursor cells known as preadipocytes. Immune cells are the second most common cell type (4).

Since the skin interfaces with the environment, it plays a variety of roles, which include providing a protective barrier against environmental factors such as bacteria or mechanical stress, regulating body temperature, and retaining water in the body (5). In addition, the skin plays a role in immunity by interacting with the immune cells within the dermis (1). Subcutaneous fat infiltrates the dermis and increased adipocytes affect the proliferation of dermal fibroblasts in obese mice (6, 7). Additionally, dermal adipocytes reportedly modulate dermal structure by regulating extracellular matrix production in dermal fibroblasts (8).

Caveolin-1 (CAV-1) is a 22 kDa membrane protein necessary for caveola formation. Caveola regulates a variety of signaling molecules and receptors that interact with the CAV-1 scaffolding domain (CSD), which corresponds to amino acid 82-101 of CAV-1 (9). CAV-1 has multiple functions, such as endocytosis, lipid homeostasis, and signal transduction; it is involved in cell proliferation and migration by associating with interacting molecules and receptors, such as Src family tyrosine kinases, integrins, epidermal growth factor receptor (EGFR), and transforming growth factor (TGF) receptors (10). Thus, numerous researchers have hypothesized the involvement of CAV-1 in the pathogenesis of inflammatory or hyperproliferative skin disorders, such as skin cancers, psoriasis, fibrosis, and wound healing. Although the contribution of CAV-1 in cell migration remains controversial depending on the cell type and the environment (11–14), CAV-1-regulated cell migration has also been reported to play a key role in skin diseases (15–19). CAV-1 involvement in skin diseases have garnered substantial attention (20–24). In this study, we focused on the latest findings on the role of CAV-1 in various skin diseases. The expression of CAV-1 in skin disorders has been investigated using several techniques (**Table 1**).

**Table 1:** Aberrant expressions of CAV-1 in human skin diseases.

Type of skin disease		Samples	Method	Results
Psoriasis		Epidermis	IHC (25–27) RT-PCR and WB (27)	Downregulated Downregulated
		Whole skin tissue	WB (26)	Downregulated
		Human PBMC	RT-PCR and WB (15)	Downregulated
		Human Monocytes	RT-PCR, WB and IF (15)	Downregulated
Systemic sclerosis (Dermal fibrosis)		Dermis	IF (28), IHC (29)	Downregulated
		Subcutaneous adipose tissue	IF (30)	Downregulated
		Human dermal fibroblasts	WB (28, 29)	Downregulated
		Human Monocytes	WB (31) and IF (30–32)	Downregulated
		Human PMNs	WB and IF (31)	Downregulated
		Human AT-MSCs	WB and IF (33)	Downregulated
Wound healing	Acute wound (Human ex vivo wound model)	Skin tissue	RT-PCR, IHC (34)	Downregulated
	Chronic wound (Diabetic foot ulcer)	Diabetic foot ulcer tissue	RT-PCR (34)	Upregulated
		plasma	ELISA (35)	Upregulated
Hypertrophic scar		Human dermal fibroblasts	RT-PCR, WB and IF (36)	Downregulated
Keloid		Human dermal fibroblasts	RT-PCR (37), WB (37, 38) and IF (38)	Downregulated
Basal cell carcinoma (BCC)		Human BCC tissue (Nodular and sclerosing type)	cDNA micro array (39)	Upregulated
		Human BCC tissue (Nodular type and infiltrative type)	IHC (40)	Downregulated
		Human BCC tissue (No pathologic type information)	IF (41)	Downregulated
Squamous cell carcinoma (SCC)		Human SCC tissue (Poorly differentiated type)	IHC (40)	Downregulated
		Human SCC tissue (No pathologic type information)	IF (41)	Downregulated
Melanoma		Serum	ELISA (42)	Upregulated
		Primary melanoma cell lines (WM-115, WM-35, MM200, WM35, WM1650, ME1402, ME10538, WM1341, WM239A)	WB (43, 44)	Upregulated (Compared to primary melanocyte)
		Metastatic melanoma cell lines (SK-MEL-28, SK-MEL-5, A-375)	WB (43)	Downregulated (compared to primary MM cell lines)
		Metastatic melanoma cell lines (WM1158, COLO793 and DX3)	WB (44)	Upregulated (compared to melanocyte)
		Tissue (primary lesions)	IHC, RT-PCR (43)	Downregulated
		Tissue (metastatic lesions)	IHC, RT-PCR (43)	Downregulated
		Tissue (vertical growth phase)	IHC (44)	Upregulated
		Tissue (metastatic lesions)	IHC (44)	Upregulated
Frontal fibrosing alopecia		Hair follicle (basal layer of outer root sheath cells)	IF (45)	Upregulated

## 2 Distribution of CAV-1 in the skin

CAV-1 is expressed in most components of the skin, including keratinocytes (46), melanocytes (47), dermal fibroblasts (28), subcutaneous white adipocytes (48) and immune cells (49). CAV-1 expression in the epidermis is most prominent in the basal and granular layers (50). CAV-1 has been suggested to regulate keratinocyte differentiation (46, 50). Decreased CAV-1 levels in keratinocytes and fibroblasts result in enhanced cell proliferation (27, 28). In melanocytes, skin pigmentation induced by UV irradiation may be modulated by CAV-1 through regulation of cyclic adenosine monophosphate (cAMP) levels (47). In adipocytes, caveolae have been reported to regulate insulin signaling (51), fatty acid transportation (52), triacylglycerol synthesis (53) and adiponectin secretion (48), indicating the involvement of CAV-1 in metabolic dysfunction.

CAV-1 is also expressed in immune cells; notably, aberrant CAV-1 expression was found in the monocytes of patients with systemic sclerosis and psoriasis and CAV-1 deficient monocytes are hypermigratory towards disease sites (15, 31).

Moreover, CAV-1 is expressed in the hair follicles (54). In C57B6 mice, CAV-1 was found in the bulge area, in which cells are multipotent and have high proliferative potential, and CAV-1 was expressed during all stages of the hair growth cycle: anagen (growing phase), catagen (transition phase), and telogen (resting phase) (54). CAV-1 expression is upregulated in the bulge area of patients with frontal fibrosing alopecia compared to healthy controls, and it is speculated that CAV-1 upregulation may contribute to the pathogenesis of alopecia (45).

While there have been no studies investigating the expression of CAV-1 in other skin appendages, sweat glands, and sebaceous glands, Kruglikov et al. speculated that CAV-1 may be involved in sebocyte function because CAV-1 interacts with TGF-β signaling and adiponectin, which regulate lipid production (55, 56).

## 3 Role of CAV-1 in skin aging

Skin aging is characterized by functional and regenerative potential losses (57). Chronologically-aged skin typically shows decreased numbers of keratinocytes, fibroblasts, and mast cells, resulting in epidermal and dermal atrophy. A significant expansion of the dermal white adipose tissue is also observed in aged skin (58, 59). During skin aging, senescent fibroblasts have impaired growth factor production and activated matrix metalloproteinases (MMPs), leading to decreased cell proliferation and enhanced degradation of the ECM, including collagen (60). Increased production of reactive oxygen species (ROS) (61), mitochondrial dysfunction (62), and DNA and oxidative damage caused by external factors (63) may contribute to age-related skin changes and pathologies.

CAV-1 contributes to cellular senescence, and its upregulation has been observed in senescent cells of several types, such as epithelial cells, fibroblasts, mesenchymal stem cells, and bone marrow stromal cells (64–66). Additionally, CAV-1 knock-out mice showed aging-related phenotypes along with mitochondrial dysfunction (67). The role of CAV-1 in cellular senescence is not fully understood and remains disputed. One study suggested that upregulation of CAV-1 by oxidative stress promotes G1 arrest and activation of the p53/p21 dependent pathway, which induces premature senescence in dermal fibroblasts (68), whereas another study showed that CAV-1 upregulation in human diploid fibroblasts inhibits cell proliferation by directly binding to growth factor receptors, causing senescence-associated growth arrest (69). Furthermore, CAV-1 silencing in senescent human diploid fibroblasts resulted in morphological changes to a young cell-like small spindle shape, probably by altering focal adhesion and actin stress fiber formation due to focal adhesion kinase and Rho family GTPase regulation (70). In contrast, CAV-1 deficiency induces cellular senescence *via* the p53/p21-dependent pathway along with mitochondrial dysfunction in several cell lines, including human diploid fibroblasts (71, 72).

The expression of CAV-1 is higher in the skin and macrophages from older mice (24 months of age) compared to that of younger mice (8-10 weeks) (73), and aged human skin (70–80 years old) has higher CAV-1 expression levels than those of teenagers (74). A negative correlation was observed between the expression levels of CAV-1 and collagen I in chronologically aged human and mouse skin, and CAV-1 silencing or depletion facilitated collagen production in dermal fibroblast (74). Senescent dermal fibroblasts induced by diabetes show CAV-1 upregulation, which may be due to oxidative stress, while inhibition of CAV-1 prevents diabetes- and oxidative stress-induced premature senescence and enhances wound healing (68).

Impaired cell proliferation is a characteristic of aged tissues. CAV-1 regulates cell proliferation in the epidermis and dermis (18, 25, 75). Keratinocyte proliferation is regulated by CAV-1, likely through the EGFR signaling pathway and Janus kinase (JAK)-signal transducer and activator of transcription (STAT) pathway (25, 75), and fibroblast cell proliferation is regulated by CAV-1, likely through phosphatidylinositol 3-kinase (PI3K)/Akt and Rho-associated kinase (ROCK) (18). Interestingly, the EGFR signaling, JAK/STAT, ROCK, and PI3K/Akt pathways possibly interact with p53-dependent pathways in other cell types (76–79), suggesting that CAV-1 may regulate cell proliferation in senescent keratinocytes and fibroblasts through these signaling pathways by interacting with p53-dependent pathways.

CAV-1 also affects the differentiation of keratinocytes, fibroblasts, and adipocytes. Sando et al. showed that CAV-1 expression is increased during the differentiation of human keratinocytes, possibly associated with protein kinase C (50). CAV-1 is also involved in human adipocyte differentiation, and was upregulated in mature adipocytes derived from human mesenchymal stem cells (hMSC) isolated from subcutaneous adipose tissue along with polymerase I and transcript release factor (PTRF). PTRF is essential for caveolae formation and is highly expressed in adipose tissue. PTRF and CAV-1 upregulation disrupted adipogenesis in a mouse adipocyte cell line (3T3-L1 cells) (80). PTRF upregulation in hMSC resulted in impaired cell proliferation and differentiation into adipocytes, and PTRF silencing promoted new adipocyte formation along with decreased p53 expression levels (81). Taken together, these results suggest that CAV-1 upregulation may play an important role in skin aging by modulating cell proliferation, differentiation, and abnormal regulation of ECM deposition through various pathways *via* crosstalk with the p53/p21 dependent pathway.

## 4 Role of CAV-1 in skin diseases

### 4.1 Role of CAV-1 in psoriasis

Psoriasis is a chronic immune-mediated inflammatory skin disease characterized by scaly skin plaques (82). Its pathogenesis is thought to be by inflammatory cytokines (e.g., tumor necrosis factor [TNF]-α, interleukin [IL]-17, IL-22, and IL-23) produced by immune cells infiltrating the dermis, leading to epidermal hyperproliferation (83). Psoriasis not only affects the skin but is also systemic, which results in an increased risk of comorbidities, such as psoriatic arthritis, cardiovascular disease, diabetes mellitus, obesity, and atherosclerosis, compared with the general population (84, 85).

We and others have reported that CAV-1 expression is decreased in the epidermis of patients with psoriasis (25–27). We found that psoriasis-related cytokines, namely IL-17, IL-22, IL-1β, and TNF-α, reduced CAV-1 expression in human keratinocytes. Reduced CAV-1 expression in keratinocytes showed enhanced cell proliferation *via* increased STAT-3 activation and enhanced cytokine production, including C-X-C chemokine ligand 8 (CXCL8), CXCL9, C-C chemokine ligand 20 (CCL20), and IL-6 (27). In patients with psoriasis, CAV-1 expression is also reduced in monocytes, and CAV-1 silencing in healthy monocytes enhances the production of cytokines such as IL-1 and IL-6 and migration towards CCL2 (15). Decreased CAV-1 expression in monocytes was reversed when patients were treated with anti-TNF-α antibodies, suggesting that psoriasis-related inflammatory cytokines also reduce CAV-1 expression in monocytes (15). Moreover, circulating monocytes in patients with psoriasis are innately polarized to the M1 phenotype, which contributes to the development of atherosclerosis (86). Silencing of CAV-1 expression in healthy monocytes prompts the polarization of macrophages to the M1 phenotype and might increase the risk of atherosclerosis by increasing macrophage oxidized low-density lipoprotein uptake (86). Leptin, an adipocyte-derived hormone, is correlated with obesity and psoriasis severity (87, 88). CAV-1 and the leptin receptor are co-localized in keratinocytes, and CAV-1 silenced human keratinocytes produce more IL-6 by co-stimulation with leptin and IL-17, suggesting that obesity deteriorates psoriasis by enhancing cytokine production in CAV-1 deficient psoriasis keratinocytes (89).

An *in vivo* model also revealed the contribution of CAV-1 to the pathogenesis of psoriasis. Imiquimod (IMQ)-induced psoriasis-like inflammation in mice showed reduced CAV-1 expression in the epidermis and monocytes, and restoration of CAV-1 function improved the severity of skin inflammation and monocyte migration into the dermis (15, 27).

This evidence suggests that CAV-1 plays an important role in the development of psoriatic skin inflammation and its comorbidities, and may accelerate chronic inflammation.

### 4.2 Role of CAV-1 in fibrotic disorders

#### 4.2.1 Systemic sclerosis

Systemic sclerosis (SSc) is an autoimmune-triggered disease characterized by vasculopathy and excessive collagen accumulation in the skin and internal organs with a high mortality (90). The ECM of the skin and internal organs promotes fibrosis (91, 92), and fibroblasts are considered effector cells (93). Several growth factors, such as TGF-β, connective tissue growth factor, and platelet-derived growth factor, can activate the pro-fibrotic response of fibroblasts (94, 95). A reduction of the thickness of dermal adipose layer is observed in SSc (96, 97), and dWAT is also reportedly involved in the development of skin fibrosis through adipocyte-myofibroblast transition (98).

Reduced CAV-1 expression has been reported in SSc-affected skin and dermal fibroblasts isolated from patients with SSc (28). TGF-β1 decreases CAV-1 expression in human skin fibroblasts in a time and dose-dependent manner (37). Restoring CAV-1 function in these fibroblasts suppresses TGF-β1-induced alpha-smooth muscle actin (α-SMA) by inhibiting the phosphorylation of Smad3, indicating that CAV-1 reduction enhances TGF-β signaling in the fibrotic response. In addition, TGF-β receptors may be directly inhibited by caveolae-mediated internalization (99).

Fibroblasts differentiated from monocytes that migrate into the dermis are also involved in SSc pathogenesis (16). Monocytes from patients with SSc have reduced CAV-1 expression, increased C-X-C chemokine receptor 4 expression, and are hypermigratory towards its receptor in lung tissue (31, 100). Circulating and dermal monocytes in patients with SSc expressing C-C chemokine receptor type 5 (CCR5) and its ligands are highly expressed in fibrotic skin tissue. Additionally, complementary CAV-1 inhibit CCR5 expressing monocyte migration, indicating that CAV-1 is involved in dermal fibrosis by modulating monocyte and fibrocyte recruitment (16).

The functional role of CAV-1 in the pathogenesis of SSc was confirmed in an *in vivo* model. CAV-1 knock-out mice showed a fibrotic skin phenotype (28, 101), and skin fibroblasts from these mice showed significantly increased expression of collagen, α-SMA, and IL-6, and decreased MMP-3 expression compared to those of wild-type mice. Restoring CAV-1 function decreased TGF-β1-induced fibrotic markers and inflammatory cytokines in fibroblasts from CAV-1 knock-out mice, and reduced fibrotic responses in a bleomycin (BLM) -induced skin fibrosis mouse model (16, 28).

#### 4.2.2 Wound healing

Wound healing is the process of skin regeneration in damaged tissue and includes increased cell proliferation, cell adhesion, and cell migration. Optimal wound healing occurs through the following processes (1): coagulation and hemostasis (2); inflammation (3); proliferation; and (4) wound remodeling with scar tissue formation (102, 103). CAV-1 contributes to this process by regulating cell proliferation and migration (34).

CAV-1 overexpression in the corneal epithelium of elderly individuals is associated with delayed wound healing (64). Jozic et al. reported an upregulation of CAV-1 in skin biopsy samples from non-healing chronic wound edges, and downregulation of CAV-1 was observed in acutely healing wounds, especially during the first 48 h of wound healing (34). They also showed that CAV-1 negatively regulates both the proliferation and migration of keratinocytes by associating with the glucocorticoid receptor and EGFR. The same authors also showed that depleting caveolae using a cholesterol-removing agent (methyl-β-cyclodextrin or mevastatin) restored EGF signaling, facilitated keratinocyte migration, and accelerated wound closure (34, 75). Further, they revealed the mechanisms of CAV-1-associated keratinocyte migration in wound healing. Increased cortisol production at wound site leads upregulation of CAV-1, which inhibit a glucocorticoid receptor repressor ArhGAP35, resulting in increased activation of Ras homolog family member A (RhoA) and diminished activation of Cell Division Cycle 42 (Cdc42) promoting keratinocyte migration and wound closure (17).

#### 4.2.3 Scarring (hypertrophic scars and keloids)

Hypertrophic scars (HTS) and keloids are thick raised scars commonly observed during the wound healing process as a result of an abnormal tissue response to injury. HTS and keloids have excess ECM components such as collagen, in the dermis and subcutaneous skin tissue and are sometimes considered to be fibroproliferative skin disorders. Both scar types have a similar disease spectrum, but HTS tends to be milder and does not expand beyond the boundaries of the original skin injury compared with keloids (104, 105). Some proinflammatory factors such as TGF-β, IL-1α, IL-1β, IL-6, and TNF-α, are upregulated in HTS and keloid tissues, making the skin more susceptible to trauma or injury (106, 107). CAV-1 expression was markedly decreased in HTS and keloid-derived human fibroblasts, and reduced CAV-1 expression mediates fibrotic responses by modulating TGF-β signaling, similar to SSc (36, 37).

Microarray results from seven Japanese patients with keloids revealed that Runt-related transcription factor 2 (RUNX2) is an upstream regulator of ECM (38). RUNX2 is a transcription factor (108) known to mediate ECM remodeling and induce aortic fibrosis (109). It regulates cell proliferation, migration, and the expression levels of ECM-related proteins and promotes apoptosis, possibly by suppressing the PI3K/Akt signaling pathway in keloid fibroblasts (18). RUNX2 expression is upregulated in keloid fibroblasts, and silencing of CAV-1 in keloid fibroblasts results in increased RUNX2 expression, which suggests that CAV1 plays a critical role in keloid formation by suppressing RUNX2 (38).

Phosphorylated CAV-1 and ROCK are upregulated in the peripheral skin tissue surrounding keloids, but not in normal skin and keloid sites, and CAV-1/ROCK expression correlates with a high inflammatory and proliferative status (110). The ROCK pathway is associated with phosphorylated CAV-1 (14), and this pathway is reported to contribute to cell proliferation (111), suggesting that the CAV-1/ROCK pathway may contribute to keloid expansion (110).

### 4.3 Role of CAV-1 in skin cancer

The function of CAV-1 as an oncogene or tumor suppressor in cancer progression remains controversial. Aberrant CAV-1 expression has been reported in several types of cancers and CAV-1 expression level sometimes depends on the tumor’s pathological subtype or clinical staging. For example, downregulation of Cav-1 has been reported in pancreatic cancer cell lines (112), primary and metastatic ovarian cancers (113), metastatic breast cancer cell lines (114), primary laryngeal squamous cell carcinoma cell lines (115), low-grade lung adenocarcinomas (116) and Barrett esophageal adenocarcinoma correlating with poor survival rates (117). In contrast, increased CAV-1 expression has been reported in moderate to severe prostate cancer (118), bladder cancer correlating with tumor grade and metastasis (119), metastatic renal cancer (120), and moderate to severe SCC of the oral cavity (moderate to severe), larynx, oropharynx, hypopharynx, esophagus (metastatic), and cervix (low grade) (121–124). Here in under, we explore the role of CAV-1 in cutaneous cancer.

#### 4.3.1 BCC

Basal cell carcinoma (BCC) is the most common skin cancer and frequently develops in areas of the skin exposed to the sun, such as the face. BCC typically grows slowly and rarely metastasizes (125).

CAV-1 expression in BCC remains controversial. Microarray profiles of 50 BCC samples in one study showed that CAV-1 gene expression was upregulated; they proposed that CAV-1 may play a dynamic role in controlling the slow progression of BCC by decreasing cellular motility since CAV-1 is known to inhibit epidermal growth factor-induced migration in other cell types (11, 39). In contrast, Gheida et al. showed a significant downregulation of CAV-1 in BCC pathological samples compared to healthy controls. Furthermore, CAV-1 expression was significantly reduced in aggressive types (micronodular, infiltrative, and metatypical BCC) compared to non-aggressive types (nodular and superficial BCC) of BCC, suggesting that CAV-1 expression levels could reflect the biological behavior of BCC and aid in the detection of high-risk patients with poor prognosis (40).

#### 4.3.2 SCC

Cutaneous SCC (cSCC) is the second most common type of non-melanoma skin cancer, after BCC. It is characterized by an abnormal, accelerated growth of squamous cells, requires surgical excision in most cases, and may lead to recurrence, metastasis, and death (126).

CAV-1 is significantly decreased in poorly differentiated types of cSCC, compared with moderately and well-differentiated types (40, 41). Trimer et al. showed that CAV-1 overexpression reduced cell growth in mouse and human SCC cell lines (127). In the same report, silencing CAV-1 in a mouse SCC cell line resulted in hyperactivation of the ERK1/2 and mitogen-activated protein kinase (MAPK) signaling pathways and increased activator protein (AP)-1 transcription factor activation. CAV-1 colocalizes and interacts with connexin 43, a known tumor suppressor (41), and it has been speculated that loss of CAV-1 affects the localization of connexin 43 and increases the activation of Ras/AP-1 signaling (40). Taken together, these findings indicate that aberrant CAV-1 expression in cSCC results in uncontrolled cell proliferation, survival, and invasion by altering multiple transduction pathways.

#### 4.3.3 Malignant melanoma

Cutaneous malignant melanoma (cMM) is a skin cancer that develops from melanocytes and melanin-producing cells. cMM is a life-threatening cancer because its proliferative and metastatic status is highly potentiated, and causes approximately 55500 deaths (0.7% of all cancer deaths) worldwide annually (128).

Nakashima et al. reported that CAV-1 overexpression in a human melanoma cell line resulted in decreased cell growth and motility (129), and other groups reported that high CAV-1 expression in stromal cells and melanoma cells was associated with longer survival in cMM patients with lymph node metastasis (130), and CAV-1 overexpression suppressed subcutaneous tumor growth (131). In contrast, some studies have reported that CAV-1 expression enhances metastasis in murine and human melanoma cell lines along with reduced E-cadherin and Rac-1 activation (131, 132). The same group reported that CAV1-enhanced melanoma cell migration, invasion, and metastasis *in vivo via* tyrosine-14 phosphorylation of CAV-1 by Src family kinases. Moreover, a transient decrease in CAV-1 phosphorylation by these kinase inhibitors prevented the early steps of lung metastasis in a murine melanoma cell line promoted by CAV-1 (132, 133). The same authors also revealed that CAV-1expressing murine melanoma cell lines showed decreased oxygen consumption, and CAV-1 expressing cells had enhanced intracellular ROS, leading to increased cell migration and invasion (19). ROS are reported to be highly expressed in cMM cells and are thought to induce DNA damage, leading to genetic alterations (134).

Aberrant CAV-1 expression has been implicated in chemotherapy efficacy. The MAPK signaling pathway plays a key role in cMM, and several BRAF and MEK inhibitors (BRAFi and MEKi, respectively) have been approved for patients with cMM harboring BRAFV600 mutations. In BRAF-mutated cMM, the efficacy of treatments targeting the MAPK pathway is high, but can decline because of the development of resistance (135). Das et al. reported the involvement of CAV-1 in BRAF inhibitor-resistant cMM cells, in which the upregulation of CAV-1 in BRAF inhibitor-resistant cMM cells led to overactivation of MAPK signaling and resulting in the suppression of BRAF inhibitors (136).

### 4.4 Role of CAV-1 in skin infection

Since caveolae directly interact with outer pathogens, such as viruses and bacteria, by modulating endocytosis (137, 138) and CAV-1 is expressed in all types of immune cells (49), CAV-1 is thought to be involved in skin infection. However, only a few studies have reported on CAV-1 and skin infections. Spaan et al. showed that CAV-1 involvement is associated with ovarian tumor deubiquitinase with linear linkage specificity (OTULIN) in severe skin necrosis after *S. aureus* infection (139). OTULIN is a linear deubiquitinase and a negative regulator of nuclear factor kB (NF-kB) signaling in the context of immunity and inflammation (140, 141), and patients with OTULIN mutations are known to be susceptible to bacterial or viral infections (139, 142). In OTULIN-deficient patients, CAV-1 accumulated in dermal fibroblasts and retained ADAM10, a cell surface receptor of α-toxin, suggesting that CAV-1 accumulation enhances the cytotoxicity of staphylococcal α-toxin (139). These results might explain why staphylococcal scalded skin syndrome (SSSS) is more severe in adults. SSSS is caused by the α -toxin produced by *S. aureus*, with blisters that spread to a large part of the body. Most patients with SSSS are infants and children, and the mortality rate is approximately 4%; however, when adults develop SSSS, the mortality rate increases to 60% (143). High CAV-1 expression in adult skin (74) may promote sensitivity to staphylococcal α-toxins. For other skin-resident bacteria, such as cutibacterium acnes and staphylococcus epidermidis, no study has reported the involvement of CAV-1 in the diseases caused by these bacteria. However, CAV-1 can possibly play some roles in infection with C. acnes or S. epidermidis since CAV-1 can be involved in the endocytosis pathway of some types of bacteria (144–146). Kruglikov et al. speculated that CAV-1 is involved in C. acnes infection (55).

(blank)For viral infections, several studies have reported on the internalization of the human papilloma virus (HPV) and herpes simplex virus 1 (HSV-1) (147–149). High-risk HPV infections, including HPV types 16, 18, and 31, are linked to Bowen’s disease (epidermal SCC in situ) (150). HPV type 31 enters keratinocytes *via* caveolae-dependent endocytosis (149, 151), suggesting that CAV-1 may affect tumor development by controlling HPV infection in keratinocytes. In contrast, HPV type 16 and HSV-1 internalization are not CAV-1 dependent (147–149).

## 5 CAV-1 as a therapeutic target in skin diseases

Various studies have suggested that the loss or gain of CAV-1 function may improve disease phenotype. The depletion of caveola accelerates wound closure by facilitating keratinocyte proliferation and migration (34, 75), and downregulation of CAV-1 in cMM enhances the efficacy of BRAF inhibitor treatment (136). Additionally, restoration of CAV-1 function may improve disorders associated with CAV-1 deficiency. To compensate for CAV-1 function, the CSD peptide (CSD, amino acids 82–101 of CAV-1) was used in several experimental settings. The CSD peptide has a sequence equivalent to CSD and is synthesized as a fusion peptide on the carboxyl-terminus of the antennapedia internalization sequence, which can cross the plasma membrane and functionally complement CAV-1 (152).

To observe the effect of the CSD peptide in psoriasis locally or systemically, we treated mice with IMQ-induced psoriasis-like inflammation by subcutaneous or intraperitoneal injections. Both CSD peptide administration routes in mice with IMQ-induced psoriasis-like inflammation showed a significantly improved phenotype and fewer infiltrating cells in the dermis compared to control mice, suggesting that CSD peptide likely improved inflammation through keratinocytes or monocytes (15, 27).

In SSc, intraperitoneal administration of CSD peptide in mice improved BLM-induced dermal fibrosis and lipodystrophy and attenuated monocyte migration into the dermis (16). In addition, CSD treatment of wild-type mice, which did not receive BLM, promoted thickening of the adipose cell layer (16).

In HTS and keloid-derived fibroblasts, CSD peptide was found to decrease ECM production in a mitogen-activated protein kinase-dependent manner and decrease TGF-β receptor I, suggesting that CSD peptide possibly ameliorates fibrosis in HTS and keloids (36, 37).

CSD subdomains (corresponding to amino acids 82-89, 88-95, and 94-101 of CSD) have also been reported to improve the skin and lung fibrosis phenotypes in BLM-treated mice. Bone marrow monocytes isolated from BLM-treated mice showed greatly enhanced migration *in vitro* towards CXCL12, and treatment with CSD and its subregions in these mice suppressed enhanced migration (153).

In conclusion, in this review, we have explored the role of CAV-1 in skin diseases. CAV-1 is involved in various skin diseases by regulating cell proliferation, migration, and enhancing proinflammatory cytokine production. Depletion or complementation of CAV-1 may improve hyperproliferative or inflammatory status, indicating that CAV-1 is a good therapeutic target in skin-related diseases.

## Author contributions

All authors listed have made a substantial, direct, and intellectual contribution to the work, and approved it for publication.

## Conflict of interest

YY declares research grants, and/or consulting fees, and/or speaker’s fees from AbbVie, Amgen, Astellas, Boehringer Ingelheim, Eisai, Eli Lilly, Janssen, Kyowa Kirin, LEO Pharma, Maruho, Mitsubishi Tanabe, Novartis, Sun Pharmaceutical Industries, Taiho Pharmaceutical, Torii Pharmaceutical, and UCB Japan.

The remaining author declares that the research was conducted in the absence of any commercial or financial relationships that could be construed as a potential conflict of interest.

## Publisher’s note

All claims expressed in this article are solely those of the authors and do not necessarily represent those of their affiliated organizations, or those of the publisher, the editors and the reviewers. Any product that may be evaluated in this article, or claim that may be made by its manufacturer, is not guaranteed or endorsed by the publisher.
